# Examination of Prokaryotic Multipartite Genome Evolution through Experimental Genome Reduction

**DOI:** 10.1371/journal.pgen.1004742

**Published:** 2014-10-23

**Authors:** George C. diCenzo, Allyson M. MacLean, Branislava Milunovic, G. Brian Golding, Turlough M. Finan

**Affiliations:** Department of Biology, McMaster University, Hamilton, Ontario, Canada; Uppsala University, Sweden

## Abstract

Many bacteria carry two or more chromosome-like replicons. This occurs in pathogens such as *Vibrio cholerea* and *Brucella abortis* as well as in many N_2_-fixing plant symbionts including all isolates of the alfalfa root-nodule bacteria *Sinorhizobium meliloti*. Understanding the evolution and role of this multipartite genome organization will provide significant insight into these important organisms; yet this knowledge remains incomplete, in part, because technical challenges of large-scale genome manipulations have limited experimental analyses. The distinct evolutionary histories and characteristics of the three replicons that constitute the *S. meliloti* genome (the chromosome (3.65 Mb), pSymA megaplasmid (1.35 Mb), and pSymB chromid (1.68 Mb)) makes this a good model to examine this topic. We transferred essential genes from pSymB into the chromosome, and constructed strains that lack pSymB as well as both pSymA and pSymB. This is the largest reduction (45.4%, 3.04 megabases, 2866 genes) of a prokaryotic genome to date and the first removal of an essential chromid. Strikingly, strains lacking pSymA and pSymB (ΔpSymAB) lost the ability to utilize 55 of 74 carbon sources and various sources of nitrogen, phosphorous and sulfur, yet the ΔpSymAB strain grew well in minimal salts media and in sterile soil. This suggests that the core chromosome is sufficient for growth in a bulk soil environment and that the pSymA and pSymB replicons carry genes with more specialized functions such as growth in the rhizosphere and interaction with the plant. These experimental data support a generalized evolutionary model, in which non-chromosomal replicons primarily carry genes with more specialized functions. These large secondary replicons increase the organism's niche range, which offsets their metabolic burden on the cell (e.g. pSymA). Subsequent co-evolution with the chromosome then leads to the formation of a chromid through the acquisition of functions core to all niches (e.g. pSymB).

## Author Summary

Rhizobia are free-living bacteria of agricultural and environmental importance that form root-nodules on leguminous plants and provide these plants with fixed nitrogen. Many of the rhizobia have a multipartite genome, as do several plant and animal pathogens. All isolates of the alfalfa symbiont, *Sinorhizobium meliloti*, carry three large replicons, the chromosome (∼3.7 Mb), pSymA megaplasmid (∼1.4 Mb), and pSymB chromid (∼1.7 Mb). To gain insight into the role and evolutionary history of these replicons, we have ‘reversed evolution’ by constructing a *S. meliloti* strain consisting solely of the chromosome and lacking the pSymB chromid and pSymA megaplasmid. As the resulting strain was viable, we could perform a detailed phenotypic analysis and these data provided significant insight into the biology and metabolism of *S. meliloti*. The data lend direct experimental evidence in understanding the evolution and role of the multipartite genome. Specifically the large secondary replicons increase the organism's niche range, and this advantage offsets the metabolic burden of these replicons on the cell. Additionally, the single-chromosome strain offers a useful platform to facilitate future forward genetic approaches to understanding and manipulating the symbiosis and plant-microbe interactions.

## Introduction

While most bacterial genomes have only a single chromosome, many are more complex and consist of two or more large replicons. Depending on their characteristics, these replicons are classified as a chromosome (largest replicon containing most of the core genes), megaplasmid (laterally acquired with a plasmid origin of replication and lacking core genes), or a chromid (displays characteristics of both chromosomes and megaplasmids) [1]. While this genome organization is most commonly found in the proteobacteria, it is by no means limited to this class [2]. Interestingly, multipartite genomes are prevalent among plant symbionts (eg. *Sinorhizobium* and *Rhizobium* species) and plant and animal pathogens (eg. *Agrobacterium*, *Vibrio*, *Burkholderia*, and *Brucella*) [1], [2]. As such, understanding the general role and evolution of these accessory replicons may provide vital insight into the biology of these organisms and possible strategies to promote or suppress these interactions.

The potential advantages of multipartite genomes imply that this genome architecture is not simply an evolutionary peculiarity. For example, the division of a genome may decrease the time required for genome replication, potentially allowing more rapid growth. Indeed, multipartite genomes are larger on average [1] and some of the fastest replicating species have divided genomes [3]. However, each replicon within a divided genome is not of equal size [3] and there is no correlation between genome size and maximal growth rate [4]. Alternatively, multipartite genomes may provide a method of controlling gene dosage and thus expression, as in *Vibrio* species [3], [5]. This can consequently result in weaker purifying selection and greater rates of evolution on the smaller replicon, as observed in *Vibrio* and *Burkholderia* [6], [7]. However, this does not hold true for slow-replicating species with a divided genome [5]. A third hypothesis is that multipartite genomes allow for additional genome expansion once the chromosome reaches its maximal size [8]. Yet, some species with multipartite genomes have primary chromosomes smaller than 2.5 Mb, while some species with a single chromosome have genomes greater than 9 Mb [9], [10]. Moreover, *Brucella* species generally have two chromosome-like replicons, except for *Brucella suis* biovar 3, which has a single chromosome equivalent in size to the total of both replicons in related strains due to integration of one replicon into the other [11], [12]. While all three of the ideas discussed above may help promote the maintenance of a divided genome architecture once established, the observations inconsistent with each suggest they are unlikely to be general driving forces for multipartite genome evolution.

An alternative hypothesis is that multipartite genomes allow for the functional division of genes onto separate replicons [13]. Several lines of evidence are consistent with this idea: uneven COG distribution between each replicon such as in *Burkholderia xenovorans* [7] and *Rhizobium etli* [14], replicon-dependent evolution in *Sinorhizobium meliloti* [15], and replicon-dependent gene regulation in *Vibrio cholerae* [16] and *S. meliloti* [17]. Furthermore, an association exists between the presence of a divided genome and an interaction with a host organism [18]. This hypothesis implies that secondary replicons are over-represented in cellular processes specific to host interaction, which, if true, should focus the genetic analyses of these processes; however, the acceptance of this idea is limited due to a paucity of experimental support [1].

*S. meliloti* is a N_2_-fixing endosymbiont of legumes, and inhabits diverse environments including bulk soil, the rhizosphere, and the legume root nodule. It is an interesting organism to study the evolution of multipartite genomes as the large 6.7 megabase (Mb) genome of the model strain Rm1021 (and the highly related strain, Rm2011) is divided into a chromosome (∼3.7 Mb), an evolutionarily old and conserved chromid (pSymB; ∼1.7 Mb), and an evolutionarily recent and variable megaplasmid (pSymA; ∼1.4 Mb) [19]–[21]. Each of these is present in all wild-type isolates [20], [21], and there is no evidence that pSymA or pSymB are naturally lost by *S. meliloti*. This indicates that each replicon is a stable and indispensible part of the genome in the natural environment. Both pSymA and pSymB encode major pathways of interaction with the plant symbiont and the environment: exopolysaccharide biosynthesis and many ABC transporters are encoded by pSymB [22], and the nodulation and nitrogen fixation genes are present on pSymA [23]. The complete removal of pSymA has been described [24], and we now report the removal of pSymB and the construction of a strain lacking both pSymA and pSymB. This reduced genome provides a novel platform to facilitate forward genetic studies of rhizobium and bacterium-plant interactions, and we employed it here to experimentally test hypotheses surrounding the evolution and role of multipartite genomes.

## Results/Discussion

*S. meliloti* forms N_2_-fixing root nodules on alfalfa and all wild-type *S. meliloti* isolates examined thus far carry replicons equivalent to pSymA and pSymB [20], [21]. Despite intensive investigation of *S. meliloti* over the past 40 years, there have been no reports of the successful removal of the pSymB chromid, with the earliest documented attempts published 25 years ago [25], [26]. The removal of pSymB as reported here was made possible through the application of several findings. First, the two essential genes (*engA* and tRNA^arg^) that are located on pSymB were integrated into the chromosome [27]. Second, an active toxin-antitoxin locus (*smb21127/smb21128*) on pSymB was removed through the introduction of a 234 kilobase deletion (ΔB180) [28]. And third, cells that failed to inherit the remaining 1.45 Mb of pSymB were recovered by selecting for the gain of a small plasmid carrying the *incα* incompatibility gene from the pSymB replication and partitioning *repABC* locus, rendering it incompatible with pSymB [29]. The latter transconjugants were obtained at a frequency of ∼10^−4^/recipient on LBmc medium containing excess cobalt, as the major *S. meliloti* cobalt uptake system (*cbtJKL*) is located on pSymB [30]. Additionally, using a similar procedure, pSymB was removed from a previously isolated strain lacking pSymA [24], resulting in cells with a genome consisting solely of the chromosome. For simplicity we refer to strains lacking pSymA, pSymB, or both, as ΔpSymA, ΔpSymB, or ΔpSymAB, respectively. The genomes of the parent and three cured strains were sequenced using an Illumina MiSeq and reads were aligned to the previously reported Rm1021 and Rm2011 genome sequences [19], [31] to confirm the absence of pSymA and/or pSymB sequences, as appropriate. The removal of pSymB is described in greater detail in the materials and methods.

Several other prokaryotic genome reduction studies have been reported in the past (e.g. *Escherichia coli* [32], *Bacillus subtilis* [33], and *Rhizobium leguminosarum* [34], [35], with the largest being a 38.9% (1.8 Mb) reduction of the *E. coli* genome [36]. The ΔpSymAB strain reported here lacks 3.04 megabases, 2866 genes, and 45.4% of the *S. meliloti* genome, and thus represents the largest genome reduction reported to date and includes the first complete removal of an essential chromid from a genome. The ΔpSymAB strain will facilitate new studies within a wide range of fields including refining the minimal symbiotic genome, general plant-microbe interactions, functional and evolutionary genomics, and biotechnology. Here, we detail phenotypic analyses of the *S. meliloti* strains lacking one or two replicons, and relate these observations to a generalized model for multipartite genome evolution.

### Nutritional requirements

Optimal growth of the ΔpSymAB strain on complex LB or TY media required cobalt [30] and calcium supplementation, while growth on minimal M9 medium is best with thiamine [37] and iron [38] addition. The affect of calcium could possibly be related to the loss of exopolysaccharide loci on pSymB. No additional nutritional requirements were identified, which was unexpected as the genome sequence indicated the asparagine biosynthesis genes to be located on pSymB [19]. Below we describe the growth of *S. meliloti* in sterile bulk soil, and interestingly, we observed that growth of the ΔpSymB and ΔpSymAB strains in this soil did not require thiamine supplementation. This indicates that thiamine biosynthesis, the sole nutrient whose biosynthesis is pSymB-dependent, is not required for growth in *S. meliloti*'s natural environment, although thiamine concentrations may limit growth in the rhizosphere [39]. Thus, very few fundamental genes are located on these replicons.

### Effects on growth

**Figure 1:**
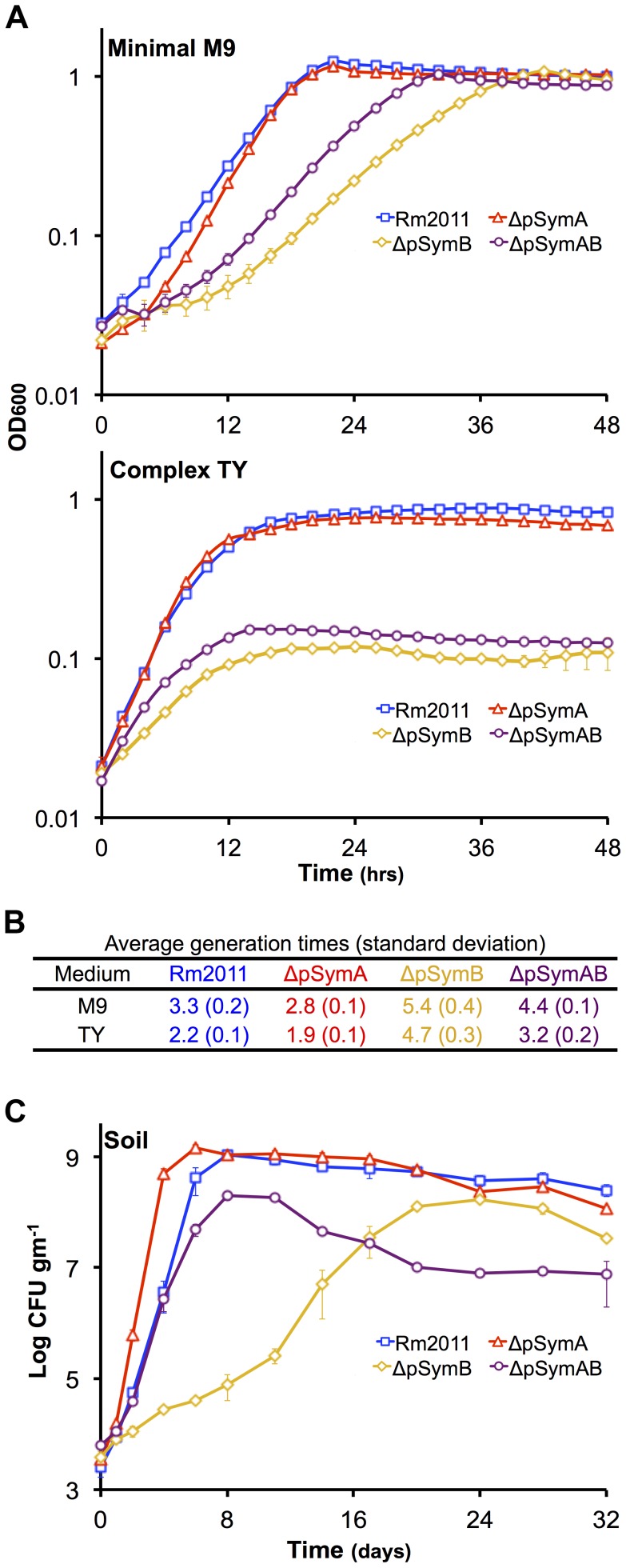
The effect of the removal of pSymA and/or pSymB on the growth of *S. meliloti*. The growth of *S. meliloti* was examined in M9 minimal medium (**A** – top panel), TY complex medium (**A** – bottom panel), or sterile bulk soil mesocosms (**C**). Data points represent averages from triplicate (**A**) or duplicate (**C**) samples. Error bars represent +/− one standard deviation from triplicate samples (**A**) or the range from duplicate samples (**C**). (**B**) Average generation times and standard deviations for each strain grown in M9 or TY media, calculated from a total of six replicates from two independent experiments. Blue – wild type; red – ΔpSymA; yellow –ΔpSymB; purple – ΔpSymAB.

**Figure 2:**
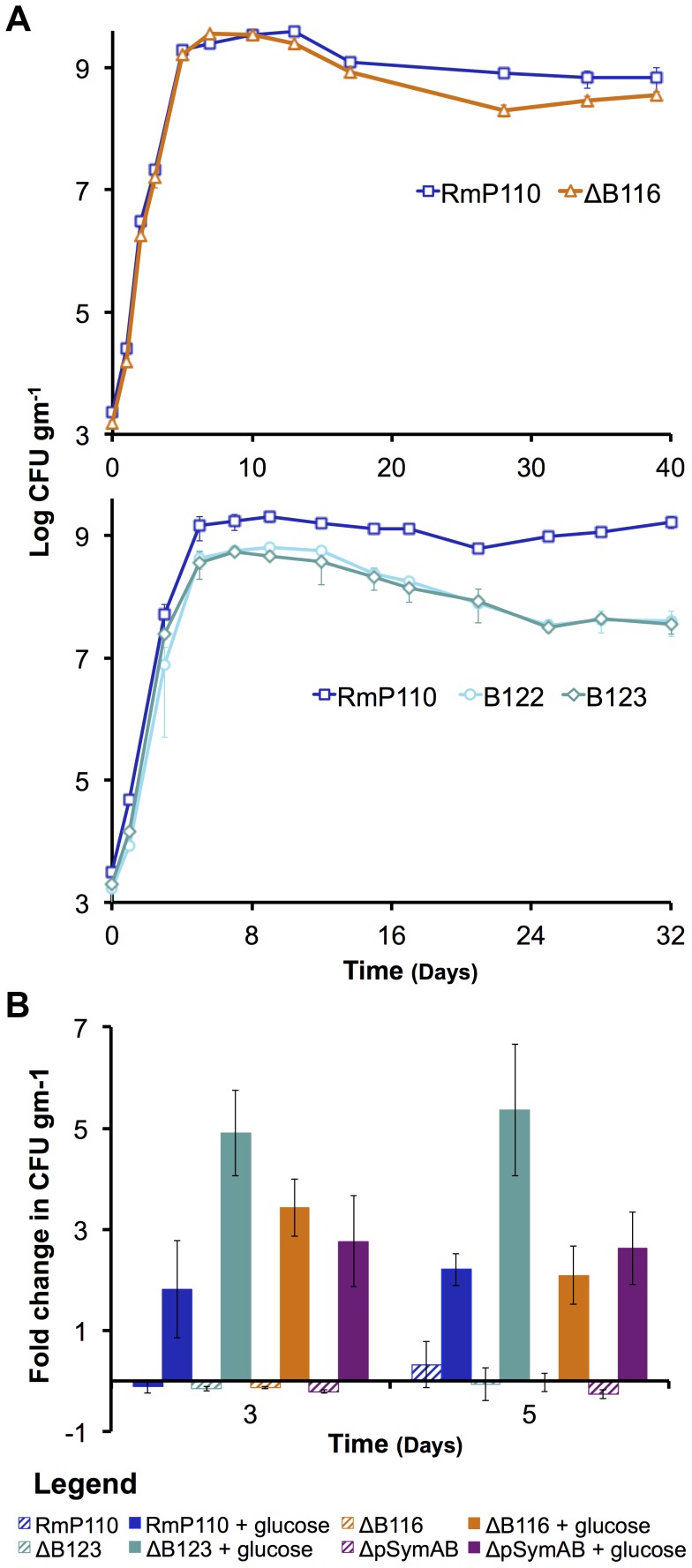
The decreased stationary phase density of strains lacking pSymB in bulk soil is due to carbon limitation and can be traced to two loci. (**A** – top panel) The strain with a deletion of B116 (orange) shows a slight, but repeatable, decrease in stationary phase density relative to the wild type (dark blue). (**A** – bottom panel) The strains with deletions of B123 (dark teal) and B122 (light teal), a sub-region of B123, show a large decrease in stationary phase density relative to the wild type (dark blue). (**B**) Stationary phase soil populations were supplemented with either 15 mM glucose (solid bars) or 5 mM NH_4_Cl, 2 mM KH_2_PO_4_, and 1 mM MgSO_4_ (striped bars). Only the addition of a carbon source (glucose) stimulated further growth for the wild type (dark blue), the ΔpSymAB strain (purple), and strains with deletions of B123 (dark teal), which includes that entire B122 region, and B116 (orange). (**A** and **B**) Data points represent the average from duplicate experiments, while error bars represent the range from duplicate samples.

Growth profiles of each strain were examined in complex and minimal media (Figures 1A, 1B, S1) by monitoring the change in OD_600_. The light scattering properties of all strains were the same as in soil mesocosm experiments described below, a 10^−4^ dilution of cell suspensions with an OD_600_ value of 1 repeatedly resulted in viable counts of 4×10^3^ CFU gm^−1^ of soil for each strain, indicating that the CFU/OD_600_ in the inoculum was 2×10^9^ for all strains (Figure 1C, 2A, 3B). Removal of both replicons led to a surprisingly small growth deficit in minimal medium, with the ΔpSymAB strain showing only a 1.37-fold slower growth rate than that of the wild type. However, a striking pattern emerged when the effect of the removal of pSymA and pSymB was examined independently: loss of the evolutionarily older pSymB resulted in a 1.6-fold slower growth rate, while loss of the evolutionarily younger pSymA led to a 1.18-fold increase in growth rate. Qualitatively similar exponential phase dynamics are observed in complex media, although a large decrease in stationary phase density is observed when the cells lack pSymB.

**Figure 3:**
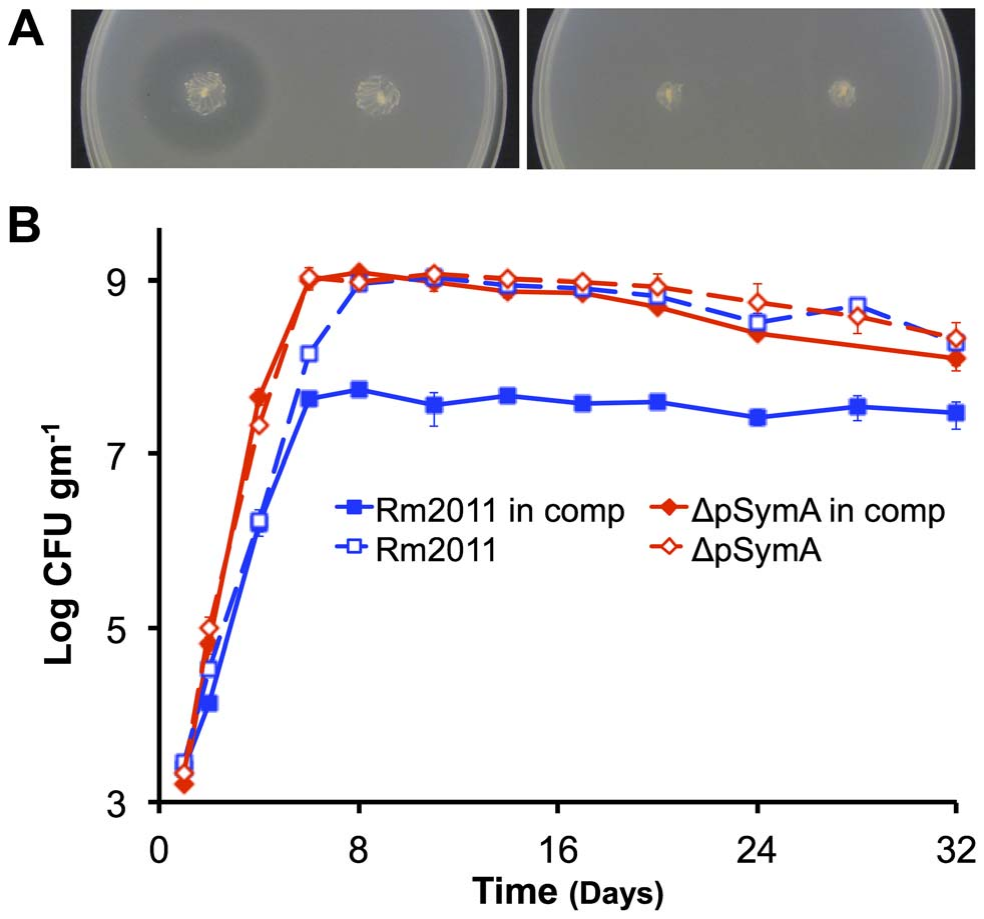
Environment specific growth inhibition by a pSymA-encoded siderophore. Growth of the ΔpSymAB strain is inhibited by a siderophore produced by the wild type (left stab) when grown in TY medium (**A** – left panel), but not if the overlay is supplemented with 150 µM FeCl_3_ (**A** – right panel). This inhibition fails to occur when the siderophore biosynthesis genes are knocked out in the wild type, as is the case in *S. meliloti* RmFL2950 (58) (right stab). (**B**) When co-inoculated in the same soil mesocosm, the ΔpSymA strain easily outcompetes the wild type, and the wild type fails to inhibit growth of the ΔpSymA strain. Data points represent averages of duplicate samples, while error bars represent the range from duplicate samples. Solid lines indicate growth pattern during co-inoculation, while dotted lines indicate growth pattern when individually inoculated. Blue – wild type; red –ΔpSymA.

Others have observed a fitness improvement following the loss of large replicons, such as a megaplasmid from *Agrobacterium tumefaciens* [40] or large virulence plasmids from pathogenic *Escherichia coli* [41]. Thus, it appears a general characteristic for large replicons is to be metabolically expensive, and that their maintenance indicates they must provide a fitness advantage to the cell not necessarily evident during laboratory growth; the symbiotic nodulation and N_2_-fixation loci on pSymA would provide such a fitness benefit. While we also expect pSymB to impose a metabolic burden on growing cells, we postulate the loss of pSymB resulted in a decreased growth rate because of the acquisition of core genes (by core genes, we mean genes that encode products that are either essential for survival or are involved in central bacterial processes) on pSymB from the chromosome (eg. *bacA*, *minCDE*, *bdhA*). It has been shown that gene transfer occurs from the primary chromosome to secondary chromosomes and chromids [8], [27]; indeed, 25–30% of genes located on pSymB that are also present in the related species *A. tumefaciens* are located on the *A. tumefaciens* circular (primary) chromosome [42]. This suggests that since their divergence, there has been significant gene transfer between the primary chromosome and secondary replicons in *S. meliloti* and *A. tumefaciens*. Furthermore, a bioinformatics approach indicated that in *Rhizobium etli* there is a correlation between the evolutionary age of a replicon and the level of functional integration with the chromosome [14]. Thus, while gene transfer from the chromosome to pSymA presumably occurs as well, the young evolutionary age of pSymA has so far precluded a significant accumulation of core genes.

### Metabolic capacity

The decreased stationary phase density of strains lacking pSymB (Figure 1B) prompted an examination of the metabolic capacity of these cells. Accordingly, wild-type *S. meliloti* and the cured derivatives were examined for the ability to grow (increase in OD600) with various sources of carbon, nitrogen, phosphorus, and sulfur. Wild-type *S. meliloti* grew on 73 carbon, 55 nitrogen, 53 phosphorus, and 20 sulfur sources (Table 1), and the removal of pSymA and particularly pSymB greatly decreased this potential (Table 1, Data sets S1, S2, S3, S4). This was most evident in carbon metabolism, as 50 of 73 carbon sources required pSymB and/or pSymA (Table 2) to be effectively utilized. As pSymA and pSymB account for 45% of the genome (20% and 25%, respectively), if the carbon transport and metabolic genes were randomly distributed throughout the genome, only 45% of the carbon sources metabolized by the wild type (equivalent 33 of the 73) should be dependent on these replicon. Thus, the data show that carbon utilization loci are over-represented on the non-chromosomal replicons (50 vs 33). Moreover, pSymB is essential for the metabolism of twice the expected number of carbon sources (36 vs 18), which is consistent with the prevalence of predicted solute ABC transporters on pSymB [22], [26], [27], [43]–[61]. Additionally, nitrogen and sulfur transport/metabolism is significantly enhanced by the presence of pSymB, although to a lesser extent than that of carbon metabolism, while phosphorus transport/metabolism is largely dependent on the chromosome (Table 1, Data sets S2, S3, S4).

**Table 1:** Nutrient sources supporting growth of *S. meliloti*.

	Number of substrate supporting growth			
Genotype	Carbon	Nitrogen	Phosphorus	Sulfur
Wild type* †	73	55	53	20
ΔpSymA	69‡	54‡	53	20
ΔpSymB	37‡	42‡	50	14
ΔpSymAB	23§	42	48§	10§

*Includes only those sources supporting good growth of the wild type.

† In several cases, the presence of either pSymA or pSymB improved growth.

‡ For both carbon and nitrogen, the usage of one source required both pSymA and pSymB.

§ In several cases, a no growth was only observed when both pSymA and pSymB were removed.

**Table 2:** Carbon sources supporting growth of *S. meliloti*.*

Sugars	
**Pentose**	**α-glucosides**
D-Arabinose†	Sucrose
D-Ribose	Maltose‡
D-Xylose‡	Turanose
*L-Arabinose*	*D-Melezitose*
*L-Lyxose*	*D-Trehalose*
**Hexose**	*Maltotriose*
D-Fructose	*Palatinose*
D-Mannose	**β-glucosides**
L-Rhamnose	Arbutin
α-D-Glucose‡	D-Cellobiose
*D-Galactose*	Gentiobiose
*D-Psicose*	Salicin
*D-Tagatose*	β-Methyl-D-Glucoside
*L-Fucose*	**α-galactoside**
*β-D-Allose*	*D-Melibiose*
**Glucose analog**	*D-Raffinose*
*3-Methyl Glucose*	*Melibionic Acid*
**Sugar phosphate**	*α-Methyl-D-Galactoside*
*D-Glucose-6-Phosphate*	**β-galactoside**
**Polyol**	*3-0-β-D-Galactopyranosyl-*
Adonitol	*D-Arabinose*
D-Arabitol	*Lactulose*
D-Mannitol	*α-D-Lactose*
D-Sorbitol	*β-Methyl-D-Galactoside*
L-Arabitol	**β-xyloside**
*Dulcitol*	β-Methyl-D-Xyloside
*Glycerol*	**Sugar amine**
*I-Erythritol*	*D-Glucosamine*
*Lactitol*	**Sugar amine derivative**
*M-Inositol*	N-Acetyl-D-Galactosamine
*Maltitol*	*N-Acetyl-D-Glucosamine*

Organic acids	
**Amino acid**	**Amino acid derivatives**
*L-Alanine*	*Glycyl-L-Proline*
*L-Arginine*	*Hydroxy-L-Proline*
*L-Aspartic Acid*	**Carboxylic Acids**
*L-Glutamic Acid*	*D-Gluconic Acid*
*L-Histidine*	*D-Glucosaminic Acid*
*L-Leucine*	*β-Hydroxy Butyric Acid*
*L-Lysine*	**Dicarboxylic Acids**
*L-Ornithine*	*D,L-Malic Acid*
*L-Proline*	*Fumaric Acid*
**γ-amino acid**	*L-Malic Acid*
*γ-Amino Butyric Acid*	*Succinic Acid*

Other	
**Nucleoside**	**Lactone**
*Uridine*	*D-Ribono-1,4-Lactone*
**Alkanolamine**	**Quarternary Ammonium**
*2-Aminoethanol*	D,L-Carnitine

*Substrate requiring pSymA and/or pSymB are indicated in italics.

† Growth on this substrate is improved by the presence of pSymA.

‡ Growth on this substrate is improved by the presence of pSymB.

### Saprophytic competence

To investigate the environmental significance of pSymA and pSymB, we developed a sterile soil mesocosm system to study the growth of wild-type *S. meliloti* and the cured derivatives (Figure 1C) (see materials and methods). In this system, the exponential growth dynamics of each strain were qualitatively similar to that in minimal medium; the loss of pSymA resulted in faster growth, the loss of pSymB impaired growth, and the removal of both resulted in an intermediate phenotype. Additionally, strains lacking pSymB showed a decreased stationary phase cell density similar to that observed in complex medium and consistent with their decreased metabolic capacity.

To identify the region(s) responsible for the growth defect associated with the removal of pSymB, a library of 14 strains in which defined regions of pSymB were deleted (representing >90% of pSymB) [28] was screened for growth in soil. None of the pSymB deletion strains showed a significant change in exponential growth dynamics, and only the loss of the two regions identified as B116 (pSymB nucleotide (nt) position 1,256,503 to 1,307,752) and B122 (nt 1,529,711–1,572,422) showed a significant and reproducible reduction in the stationary phase density in soil (Figure 2A). To investigate whether carbon availability was a growth-limiting factor in the soil, 15 mM glucose was added to stationary phase soil cultures of the wild type, ΔpSymAB strain, and strains with deletions of either the B116 or B122 regions. Viable cell counts following 3 and 5 days of incubation showed that glucose stimulated growth of all four strains, whereas no growth stimulation was observed following supplementation with nitrogen, phosphorus, and sulfur (Figure 2B). Thus, the availability of a usable carbon source appears to be a major factor limiting stationary phase growth for all strains in the soil mesocosms.

The deletion of B116 results in a 2 fold decrease in viable cell density in bulk soil, and the removal of B122 results in a 5–25 fold reduction (Figure 2A). While we have not confirmed which genes within these regions are responsible for the observed phenotype, we note that the B122 region includes genes (*bhbA-D*) involved in metabolism of the carbon storage compound poly-3-hydroxybutyrate [62], while half of the B116 region spans a DNA fragment known to have translocated to pSymB from the chromosome in a *S. meliloti* ancestor [27]. As the stationary phase defect associated with the loss of both B116 and B122 is related to decreased carbon metabolic abilities (Figure 2B), it is reasonable to assume a multiplicative effect if both B116 and B122 are removed simultaneously, which would be a 10–50 fold decrease in stationary phase density. In fact, this is highly consistent with the observed stationary phase reduction of the ΔpSymB and ΔpSymAB strains (Figure 1C). Thus, we propose that the stationary phase defect associated with the removal of pSymB may be attributed predominately, if not entirely, to the loss of genes within these two regions.

In summary, the growth rate of *S. meliloti* in soil appears to be positively impacted by the removal of pSymA, but negatively impacted by the removal of pSymB, likely for the reasons discussed previously (see ‘effects on growth’). On the other hand, the evidence shows that few pSymA- or pSymB-encoded metabolic capabilities are biologically necessary during growth of *S. meliloti* in sterile bulk soil. Thus, we wondered what evolutionary pressures maintain these metabolic capabilities. Slater *et al.* [8] presented strong bioinformatics evidence suggesting the common ancestor of the *Rhizobiales* order contained a single chromosome, and that this species captured a *repABC* plasmid (which they referred to as the ITR) that has evolved into secondary chromosomes or chromids in many modern day *Rhizobiales* (eg. pSymB in *S. meliloti*, and the second chromosome of *Agrobacterium* species). The presence of exopolysaccharide biosynthetic genes, which facilitates a strong plant-microbe interaction [18], on pSymB [22] and the second chromosome of *Agrobacterium* species [8] suggest that these genes may have originated on the ITR. Furthermore, phylogenetic studies have concluded that the evolution of an association with plants was associated with a large increase in solute, and particularly sugar, transporters [63], [64]. Indeed, *S. meliloti* is capable of using a broad range of carbon sources for growth, and these functions are significantly over-represented on pSymB. Consequently, we suspect that an early plasmid derived from the ITR allowed improved colonization of the rhizosphere, leading to a selection for new genes specific to growth in this novel niche. Unlike plasmids, large rearrangements of bacterial chromosomes are generally selected against [8], [65], thus the subsequent genome expansion occurred primarily with the ITR-derived plasmid, resulting in a replicon specialized for growth in the rhizosphere. While the fitness advantage provided by pSymB in the rhizosphere was not directly assessed here, we note that many of the carbon sources unable to support growth of the ΔpSymAB strain are indeed present in the rhizosphere (e.g. organic acids, galactosides, and several polyols and sugars [66]–[68] (Table 2).

### Competitive phenotype

In natural environments, microorganisms are found as mixed populations and compete with each other for available resources. We therefore wished to examine whether pSymA and pSymB influences the competitive fitness of *S. meliloti*. Interestingly, in an agar plate assay, growth of the wild-type *S. meliloti* was found to inhibit the growth of strains lacking pSymA (Figures 3A, S2), but not the ΔpSymB strain (Figure S2). While such inhibition was observed previously [69], the nature of the inhibition was not identified. To identify loci responsible for this phenotype, we analyzed a library of strains, in which defined regions of pSymA were deleted [28], for inhibition by the wild type. This screen identified a 64 kilobase region (A133) whose loss confers sensitivity to the inhibition by the wild type. This region encodes siderophore biosynthetic (*rhbA-F*) and uptake (*rhtA, rhtX*) genes [70], [71], and subsequent mutant analysis revealed that simply disrupting the siderophore uptake genes conferred sensitivity to inhibition by the wild type (Figure S2), while disrupting the biosynthetic genes in the wild-type background precluded inhibition of the ΔpSymAB strain (Figure 3A). Furthermore, no inhibition was observed in the presence of excess iron (Figure 3A). Taken together, these analyses revealed that inhibition was mediated through the siderophore sequestering environmental iron from the ΔpSymA or ΔpSymAB strains (Figures 3A, S2).

The effect of this siderophore during soil growth was examined through co-inoculation of the wild type and the ΔpSymA strain in the same soil mesocosm (Figure 3B). Consistent with carbon being the growth-limiting nutrient and available iron being in excess, the presence of the wild type did not impact the growth of the ΔpSymA strain, and the ΔpSymA strain easily outcompeted the wild type. In line with this result, Loper and Henkels [72] previously reported that the *Pseudomonas fluorescens* siderophore was not expressed during growth in bulk soil. However, the synthesis/uptake of a siderophore may impact fitness in the rhizosphere [72] and possibly affect symbiosis [70].

**Figure 4:**
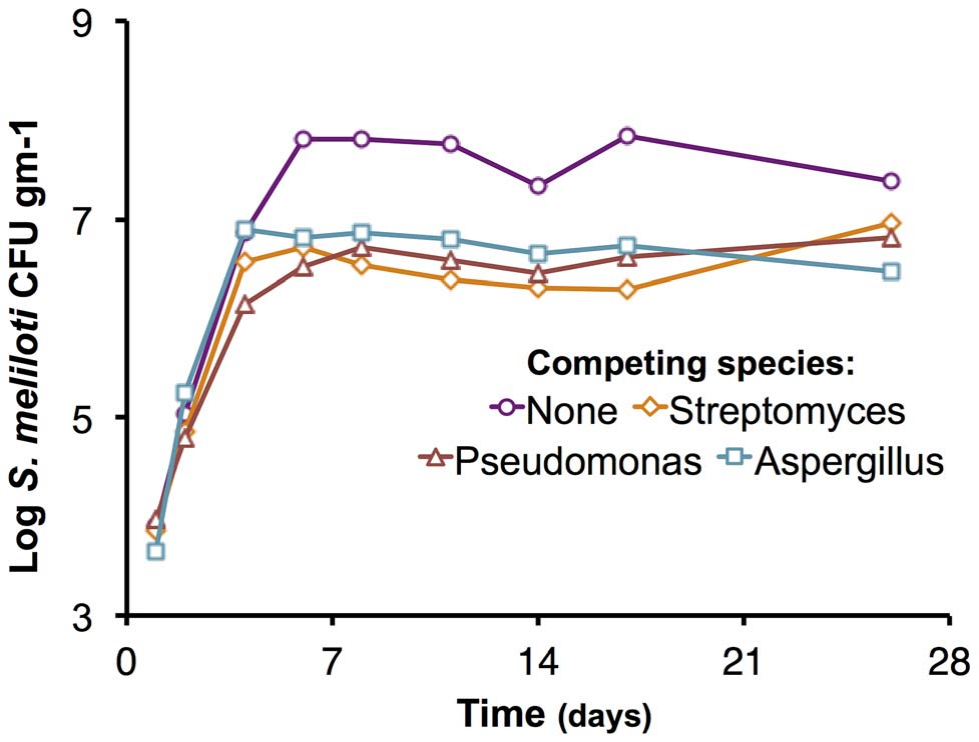
Effect of competing species on the growth of the *S. meliloti* ΔpSymAB strain in bulk soil mesocosms. The *S. meliloti* ΔpSymAB strain was grown in bulk soil mesocosms in the presence of either an *Aspergillus* species, *Pseudomonas syringae*, or *Streptomyces ceolicolor* and the growth of the ΔpSymAB strain was examined. The decreased stationary phase density during competition is presumably reflective of competition for nutrients and the reduced availability of nutrients due to usage by the competitor. On the other hand, there is a relative lack of effect on the early exponential growth of the ΔpSymAB strain, and it is able to establish a stable stationary phase population in the presence of the competing species. See materials and methods for details on experimental set-up. Purple – ΔpSymAB alone; teal – ΔpSymAB with a soil-isolated *Aspergillus* species; brown – ΔpSymAB with *P. syringae*; orange – ΔpSymAB with *S. ceolicolor*. Data points represent single values.

In addition to intra-species competition, inter-species competition is a major fitness determinant. We assessed the growth of the ΔpSymAB strain in the presence of three competing species: *Pseudomonas syringae*, *Streptomyces ceolicolor*, and a soil-isolated *Aspergillus* species (Figure 4). The early exponential growth of the *S. meliloti* strain was not adversely impacted by any of the competing species, and the ΔpSymAB strain was able to establish a stable population in the presence of these species over the course of the 26-day assay. However, we observed that the maximum cell density attained by the ΔpSymAB strain was decreased ∼10–20 fold when co-inoculated with a competitor, which may be attributed to inter-species competition for common nutrients and energy sources. As a whole, our data nonetheless suggest that neither pSymA nor pSymB are required for *S. meliloti* to effectively establish a long-term population or compete for resources with other species, and their loss does not render *S. meliloti* susceptible to killing by these species.

### Model of multipartite genome evolution

There are two general scenarios for the evolution of multipartite genome evolution. The schism hypothesis suggests that second chromosomes or chromids result from the split of an ancestral chromosome into two [18]. This has been suggested to have occurred in *Rhodobacter sphaeroides* [73]. Alternatively, the plasmid hypothesis suggests chromids result from the capture of a megaplasmid that subsequently acquires core genes from the chromosome [18]. The often-observed bias for essential genes to be located on one chromosome suggests that the plasmid hypothesis is more generally applicable [18], and evidence suggests that the plasmid hypothesis is true in the case of *Vibrio*, *Agrobacterium*, *Rhizobium*, and *Sinorhizobium*, among others [2], [8], [13], [18], [21].

**Figure 5:**
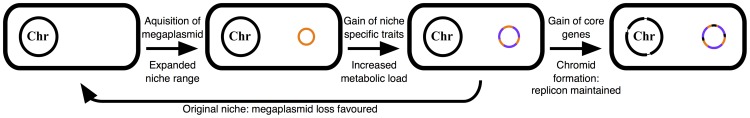
Schematic illustrating the described model of multipartite genome evolution and chromid formation. The acquisition of a megaplasmid (orange) expands the niche range of the cell. Subsequently, this replicon accumulates horizontally acquired genes that provide a fitness advantage in this novel environment (purple). This results in a large metabolic load being associated with the megaplasmid, and its loss is favoured in the cell's original niche. However, gene transfer from the chromosome (black) renders the megaplasmid (now a chromid) indespensible in all environments. See the text ‘model of multipartite genome evolution’ for additional details.

Several hypotheses exist about the function of multipartite genomes, and the evolution of multipartite genomes through the plasmid hypothesis; however, little experimental evidence has previously been reported to support these ideas. The presence of two replicons with distinct evolutionary histories (ie. pSymA was a much more recent addition to the genome than pSymB) and characteristics (ie. megaplasmid vs chromid), and the presence of strains lacking one or both of these replicons makes *S. meliloti* an ideal system in which to experimentally develop a model describing the evolution of multipartite genomes. In the proposed model (Figure 5), as a first step a host cell captures a plasmid that encodes genetic determinants allowing the cell to occupy a novel niche. Inhabiting this new environment puts an evolutionary pressure on the cell to obtain additional genetic material that provides a fitness benefit unique to this location. As genetic rearrangements of bacterial chromosomes are generally associated with a fitness cost [65], this new genetic material is disproportionately acquired by the plasmid, resulting in a plasmid specialized for a specific niche. As plasmids are mobile elements, this enrichment of niche-specific traits is advantageous as it would promote plasmid retention following transfer to a new unichromosomal organism. From the host's view, while this plasmid is valuable in the new niche, its specialized nature means it provides little advantage in the original environment and is in fact a fitness burden due to its metabolic load. In *S. meliloti*, pSymA represents an example of a plasmid that encodes functions essential to a specialized niche (forming N_2_-fixing root nodules with legumes) and yet imposes a fitness cost to cells growing in the species original environment (bulk soil). Thus, strains lacking pSymA grow more rapidly and outcompete wild-type *S. meliloti* in bulk soil (Figures 1, 3B), although ΔpSymA strains are unable to form root nodules [23], [38] and growth of the ΔpSymA strains may be inhibited by the wild type in specific environments (Figure 3A).

Over time, random translocations from the chromosome to a resident plasmid would result in the formation of a chromid, leading to an evolutionary pressure to maintain the chromid in all environments, including the species original niche where the loss of the replicon would otherwise be favored. pSymB has had a long association with the *S. meliloti* lineage and during this time has acquired core elements from the chromosome [8], [27], [42]. Loss of this replicon adversely affects the growth of *S. meliloti* in bulk soil (Figure 1C) despite the reduced metabolic demand of no longer maintaining the chromid. However, the many metabolic functions dependent on pSymB largely appear to not be necessary for growth in bulk soil, and may be more relevant during growth in the rhizosphere, consistent with a niche-specialized role of this replicon. Overall, the phenotypic data reported here support a model where environmental specialization is a general driving force for multipartite genome evolution, with secondary replicons being enriched for functions unique to the new environment. Indeed, previous comparative genomics analysis [1], [8], [15] presented evidence consistent with many of the core postulates of this model that were derived through experimental examination.

While this model addresses the evolution and primary role of secondary replicons, it is still unclear as to why this genome architecture persists, and why secondary replicons do not integrate into the chromosome. Integration has been postulated to have occurred in *Mesorhizobium* and *Bradyrhizobium* [8], which carry a single large chromosome, despite having similar lifestyles to *Sinorhizobium* and *Rhizobium*, which have divided genomes. While it is possible that the presence of a divided genome is an evolutionarily transient event, this seems unlikely. As discussed in the introduction, several advantages have been ascribed to the presence of a multipartite genome that may promote its maintenance. Indeed, *S. meliloti* strains that carry all three replicons recombined into one show a growth defect [74], illustrating how genome structure and not just gene content affects the cell's phenotype. There may also be constraints on the ability of a chromid or megaplasmid to recombine into the chromosome. The origin and terminus of replication separate bacterial chromosomes into subdivisions that tend to be equal in size. Large insertions, such as the integration of a secondary replicon into the primary chromosome, would disrupt this balance and thus be unfavourable [75]. Additionally, it has been suggested that there is an upper size limit of bacterial chromosomes, which could potentially preclude the integration of a large replicon into the chromosome [8]. Finally, plasmids, being mobile elements, can move into naïve cells, leading to further propagation of their DNA. As such, the fitness of the plasmid would be reduced following recombination into the main chromosome.

## Materials and Methods

Except for the strains and plasmids contructed in this study, all other strains and plasmids have been previously described [27]–[29], [37], [76]–[78] and are listed in Table S1.

### Growth conditions

Complex media included LB (10 gm/L tryptone, 5 gm/L yeast extract, 5 gm/L sodium chloride), LBmc (LB with 2.5 mM MgSO_4_ 2.5 mM CaCl_2_), and TY (5 gm/L tryptone, 2.5 gm/L yeast extract, 10 mM CaCl_2_). For growth of *S. meliloti*, complex media was supplemented with 2 µM CoCl_2_. Minimal media included M9 (41 mM Na_2_HPO_4_, 22 mM KH_2_PO_4_, 8.6 mM NaCl, 18.7 mM NH_4_Cl, 4.1 µM biotin, 42 nM CoCl_2_, 1 mM MgSO_4_, 0.25 mM CaCl_2_, 38 µM FeCl_3_, 5 µM thiamine-HCl, 10 mM sucrose) and a 4-morpholinepropanesulfonic acid (MOPS) buffered medium (M9 with the phosphate buffer replaced with 40 mM MOPS and 20 mM KOH, with 2 mM KH_2_PO_4_). For the phenotype macroarray analysis, cultures were grown in M9 medium for the carbon and sulfur analyses, while strains where grown in MOPS medium for the nitrogen and phosphorus analyses. Additionally, the concentration of biotin was reduced to 40 nM for the analysis of sulfur metabolism. Unless stated otherwise, antibiotics were added to the following concentrations (µg/mL) for *S. meliloti* (*E. coli*), when appropriate: streptomycin 200 (N/A), spectinomycin 100 (100), tetracycline 5 (5), gentamicin 60 (20), neomycin 200 (N/A), kanamycin N/A (25), and chloramphenicol N/A (5). Antibiotic concentrations were halved for liquid media. *S. meliloti* was grown at 30°C and *E. coli* was grown at 37°C.

### Genetic techniques

Common genetic techniques and manipulations were performed as previously described [76], [79], [80].

### Growth curves

Overnight cultures were washed, resuspended, and diluted in fresh media. 150 µL of diluted cultures (OD_600_∼0.05, measured with a 1 cm wavelength) were inoculated into 96-well plates, with each strain done in triplicate. The edges of the 96-well plates were taped to prevent moisture loss and the 96-well plates were incubated in a Tecan Safire for 48 hours at 30°C (+/−1°C) with shaking. OD_600_ measurements were taken every 15 minutes. A Perl script was written to calculate averages, standard deviations, and generation times.

### Phenotype macroarray

Phenotype macroarrays were performed in Biolog plates (PM1, PM2A, PM3B, PM4A). Overnight cultures were washed, resuspended, and starved overnight in media free of the appropriate nutrient. Starved cultures were washed, resuspended, and diluted in fresh media, then 100 µL was inoculated into each well of the Biolog plates. Plates were incubated at 30°C for 5–7 days in a SteadyShake 757 Benchtop Incubator Shaker (Amerex Instruments, Inc.), with OD_600_ readings taken every 12–24 hours.

### Soil preparation

In 2007, a 40 kg soil sample was obtained from an alfalfa field within a dairy farm near Guelph, Ontario, Canada, which does not apply pesticides, fertilizers, or herbicides. Large materials were manually removed, and following 9 days of drying, the soil was passed through a sieve to remove fragments larger than 2 mm. The soil was heat sealed in polyethylene freezer bags (FoodSaver; Jarden Corporation) as 100–300 gm samples. Soil samples were subjected to γ-irradiation (using ^6^°Co as a source) at the McMaster University Nuclear Reactor with a final dosage of 25.0 kGy (over a period of 54.3 hrs). As subsequent testing revealed the soil was not sterile, a second round of γ–irradiation at a final dosage of 42.3 kGy was performed, and stored at −20°C until use. However, as a *Dienococcus* species still remained viable, soil samples were autoclaved once (123°C; 17 psig; 20 minutes) within a few days of beginning each growth assay. A chemical analysis of the soil was performed by the University of Guelph Laboratory Services Agricultural and Food Laboratory (Guelph, Ontario, Canada), and the results are presented in Table S2.

### Soil growth protocol

47.62 gm (40 gm dry weight) of γ–irradiated soil was added to 500 mL screw-capped glass bottles (Gibco), autoclaved, and allowed to cool. Within a few days, *S. meliloti* strains were grown in LBmc or TY and cells were washed once with 0.85% NaCl and three times with de-ionized, autoclaved water (ddH_2_O). Cells were resupended in ddH_2_O, adjusted to an OD_600_ of 1 and serial diluted to 10^−4^, which equals approximately 2×10^5^ CFU/mL. 1 mL of this dilution, together with an additional 1.38 mL ddH_2_O, was added to each mesocosm. The resulting mesocosm contained 50 gm soil (40 gm dry weight a 20% moisture (wt/vol)), and ∼4×10^3^ CFU gm^−1^. Soil mesocosms were incubated at room temperature (22°C+/−2°C) in the dark, and soil moisture content was maintained by the addition of ddH_2_O every one to two weeks (at the rate of 48 µL per day).

To determine cell density, 0.62 gm samples were removed from each mesocosm into a 2 mL Eppendorf tube in a sterile environment. 1 mL of 0.85% NaCl was added to each tube and cells were re-suspended with vigorous vortexing. Soil particles were pelleted by vortexing for 1 minute at 60 *g*. The supernatant was serial diluted and plated on LB or LBmc to determine CFU gm^−1^.

Throughout co-inoculation experiments, when plating for CFU gm^−1^, dilutions were plated on non-selective and selective media. When wild-type *S. meliloti* and the ΔpSymA strain were co-inoculated, each strain was inoculated to ∼2×10^3^ CFU gm^−1^, and strains were differentiated based on growth with 10 mM trigonelline as the sole carbon source as only the wild type will grow. For co-inoculation of *S. meliloti* with *P. syringae*, *S. meliloti* was inoculated to ∼2×10^3^ CFU gm^−1^ while *P. syringae* was inoculated to ∼8×10^2^ CFU gm^−1^, and CFU gm^−1^ was determined by plating on LB with streptomycin (*S. meliloti*) or LB with 20 µg/mL rifampicin (*P. syringae*). When co-inoculated with *S. ceolicolor*, *S. meliloti* was inoculated to ∼2×10^3^ CFU gm^−1^ while *S. ceolicolor* was inoculated with 2×10^3^ spores gm^−1^, and *S. meliloti* was selected for with streptomycin. Co-inoculation with *Aspergillus* was initiated with ∼4×10^3^ CFU gm^−1^ of *S. meliloti* and ∼8×10^2^ spores gm^−1^ of *Aspergillus*, and CFU gm^−1^ of *S. meliloti* was determined on LB with 100 µg/mL cycloheximide.

### Isolation of a soil *Aspergillus* species

A 0.45 gm sample of non-sterilized soil used for the soil growth assays was vigorously vortexed in 1 mL 0.85% saline, and dilutions were plated on YPD medium (10 gm/L yeast extract, 20 gm/L peptone, 20 gm/L dextrose, 15 gm/L) with 50 µg/mL chloramphenicol. Based on morphology, an *Aspergillus* species was identified and streak purified on YPD. The *Aspergillus* was sporulated on LCA medium [81] for eight days at 30°C, and spores were re-suspended in PBS (0.8% NaCl, 0.02% KCl, 0.144% Na_2_HPO_4_, 0.024% KH_2_PO_4_) with 100 µg/mL streptomycin. Spores/mL were determined by counting spores with a hemacytometer.

### Bacteriocin assay

This assay was performed essentially as described previously [69], [82]. Strains being tested for bacteriocin production were stabbed into TY agar plates and incubated at 30°C overnight. The next day, surface growth of the producer was largely removed using a sterile toothpick. Overnight cultures of strains being tested for bacteriocin sensitivity were diluted to an OD_600_∼0.01 in TY, and 1 mL was mixed with 5 mL of TY with 240 µg/mL streptomycin or spectinomycin and 0.6% agar (giving a final concentration of Sm^200^ or Sp^200^ and 0.5% agar), and all 6 mL were poured onto the plates with the stabbed producers. The inclusion of streptomycin or spectinomycin was to prevent the producer from growing into the soft agar overlay. Plates were incubated at 30°C for two nights, following which zones of clearance were identified. When applicable, 150 µM FeCl_3_ was added to the soft agar overlay.

### Removal of pSymB

Previous work has indicated that there are only two single copy essential genes outside of the chromosome (*engA* and tRNA^arg^), both located on pSymB [27], [28]. Previously, these two essential genes were integrated into the chromosome [27]. However, this integration included a neomycin resistance marker, and we wished to use neomycin as a selective marker during the process of removing pSymB. Thus, it was necessary to begin by constructing a neomycin sensitive integration of the essential genes into the chromosome.

Based on how the integration was performed, there are two possible orientations of the genes following integration (Figures S3A, S3B), and using PCR we determined that the construct integrated as seen in Figure S3B (RmP2686). Using the same procedure as previously followed [27], we isolated a second strain with the orientation illustrated in Figure S3A (RmP2711). The insertion in RmP2711 was transduced into a *metH*::Tn5-B20 strain selecting for spectinomycin resistance; *metH* is located ∼5 kilobases upstream of the *engA*/tRNA insertion site (Figure S3C). The resulting strain was the recipient in a transduction with a phage lysate prepared from RmP2686 (Figure S3D). Colonies were selected for based on a MetH^+^ phenotype on minimal medium and screened for Nm resistance. Following the isolation of a neomycin sensitive colony, PCR was used to confirm that the genetic organization of the insertion was as expected (Figure S3E). The insertion in this final strain, RmP2719, is stable and is neomycin sensitive.

The two-gene operon, *smb21127/smb21128* (pSymB nt: 766,498–767,430), functions as an active toxin-antitoxin locus, although it is possible to delete this system with a low frequency [28]. Therefore, the deletion ΔB180 (pSymB nt: 635,940 nt–869,645) was transduced into *S. meliloti* strain Rm2011, selecting for neomycin resistance. Subsequently, the chromosomal integration of *engA* and tRNA^arg^ was transduced into this strain, selecting for spectinomycin resistance. The resulting strain, RmP3005 carries the essential pSymB genes on the chromosome as well as a 234 kilobase deletion that removed the only known active toxin-antitoxin system on this replicon.

The replication and segregation machinery of pSymB is encoded by the *repABC* operon [29]. An incompatibility factor, *incA*, is encoded within the *repB* and *repC* intergenic region; thus, pSymB cannot be stably co-inherited with another replicon carrying an exact copy of *incA* [29]. Thus, pTH1414 (pOT1 carrying the pSymB *incA* region) [29] was introduced into *S. meliloti* RmP3005, and streptomycin/gentamicin resistant colonies were selected for on LB supplemented with 2 µM cobalt chloride to compensate for the loss of the major *S. meliloti* cobalt uptake ABC transporter (CbtJKL), which is pSymB-encoded [30]. Recovered colonies were streak purified, and initially the inability to amplify six pSymB fragments using PCR was evidence that pSymB was indeed lost. One colony was inoculated in LBmc broth, serial diluted and plated on LB, and colonies were screened for loss of pTH1414 by patching for gentamicin sensitivity. A gentamicin sensitive colony was purified and stored as *S. meliloti* RmP3009.

In order to construct the strain lacking both pSymA and pSymB, the same procedure was followed as above, with two modifications. The starting strain lacked pSymA [24]. Additionally, the chromosomal integration of *engA* and tRNA^arg^ from *S. meliloti* RmP2719 was transduced into this strain prior to the transduction of ΔB180. Following the removal of pSymB using incompatibility, and the subsequent loss of pTH1414, the resulting strain was frozen as *S. meliloti* RmP2917, which lacks both pSymA and pSymB.

## Supporting Information

Data set S1 Carbon phenotype macroarray results. Results of the carbon utilization phenotype macroarray experiments. The ability of each strain to grow (Yes) or not grow (No) with each tested carbon source is indicated.
(XLSX)

Data set S2 Nitrogen phenotype macroarray results. Results of the nitrogen utilization phenotype macroarray experiments. The ability of each strain to grow (Yes) or not grow (No) with each tested nitrogen source is indicated.
(XLSX)

Data set S3 Phosphorus phenotype macroarray results. Results of the phosphorus utilization phenotype macroarray experiments. The ability of each strain to grow (Yes) or not grow (No) with each tested phosphorus source is indicated.
(XLSX)

Data set S4 Sulfur phenotype macroarray results. Results of the sulfur utilization phenotype macroarray experiments. The ability of each strain to grow (Yes) or not grow (No) with each tested sulfur source is indicated.
(XLSX)

Figure S1 The effect of the removal of pSymA and/or pSymB on the growth of *S. meliloti*. (**A**) Growth curves of the wild type and replicon cured strains in LBmc. Data points represent averages from triplicate, and error bars represent +/− one standard deviation from triplicate samples. (**B**) Average generation times and standard deviations for each strain grown in LBmc medium, calculated from a total of six replicates from two independent experiments. Blue – wild type Rm2011; red – ΔpSymA; yellow – ΔpSymB; purple – ΔpSymAB.
(TIFF)

Figure S2 Images showing the bacteriocin-like effect of the pSymA-encoded siderophore. In all images, the stabbed strain is the wild-type *S. meliloti* Rm5000 (60), which is a rifampicin resistant and streptomycin sensitive derivative of the same nodule isolate of *S. meliloti* Rm2011 (streptomycin resistant). The wild type is able to inhibit the growth of strains lacking pSymA, but not a strain lacking just pSymB. The sensitivity of the pSymA cured strain is conferred by the inability to uptake the siderophore, as is seen by the sensitivity of *S. meliloti* RmFL2878 (*rhtA*::pTH1522), which is a siderophore uptake mutant (59).
(TIFF)

Figure S3 A diagrammatic representation of how the neomycin sensitive integration of the pSymB essential genes into the chromosome was constructed. (**A**) and (**B**) represent the two possible genetic organizations following integration into the chromosome, while (**E**) represents the final genetic organization. The arrows represent the approximate location of primers able to differentiate between each of the three organizations; the open-ended arrows will amplify a product in (**B**) and (**E**), while the close-ended arrows will amplify a product in (**A**) and (**E**). (**C**) and (**D**) illustrate the two transductions involved in the creation of a neomycin sensitive integration. The Tn5 insertion in *metH* is a loss-of-function mutation, rendering the strain unable to grow on minimal medium not supplemented with methionine. Diagrams are partially to scale. The *attR* sequence is indicated by the black arrowheads, the *attL* sequence by the light gray arrowheads, and the *attP* sequence by the white arrowheads. 1 – *pmi*; 2 – *Y03111*; 3 – *mak*; 4 – tRNA^CCT^; 5 – *Y03108*; 6 – *Y03107*; 7 – *Y03106*; 8 – *dxr*.
(TIFF)

Table S1 Bacterial strains and plasmids.
(DOCX)

Table S2 Physiochemical properties of the soil used in this study.
(DOCX)
