# Supplementary figures and images for: Examination of Prokaryotic Multipartite Genome Evolution through Experimental Genome Reduction
Source: PLoS Genet. 2014 Oct 23;10(10):e1004742. doi: 10.1371/journal.pgen.1004742 (PMC4207669; doi:10.1371/journal.pgen.1004742)

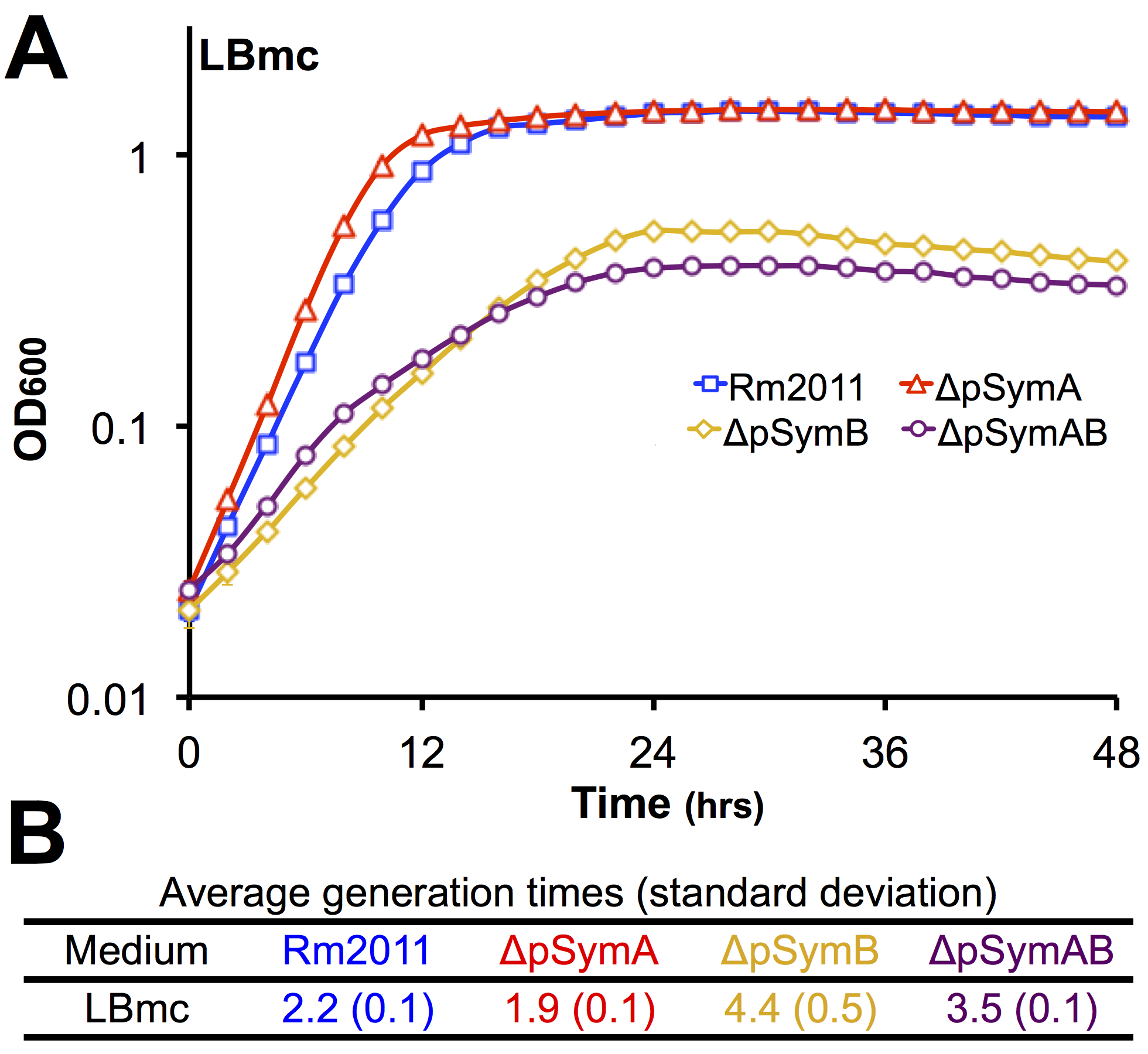

Supplement: Figure S1 — The effect of the removal of pSymA and/or pSymB on the growth of S. meliloti. (A) Growth curves of the wild type and replicon cured strains in LBmc. Data points represent averages from triplicate, and error bars represent +/− one standard deviation from triplicate samples. (B) Average generation times and standard deviations for each strain grown in LBmc medium, calculated from a total of six replicates from two independent experiments. Blue – wild type Rm2011; red – ΔpSymA; yellow – ΔpSymB; purple – ΔpSymAB. (TIFF) [file pgen.1004742.s005.tiff]

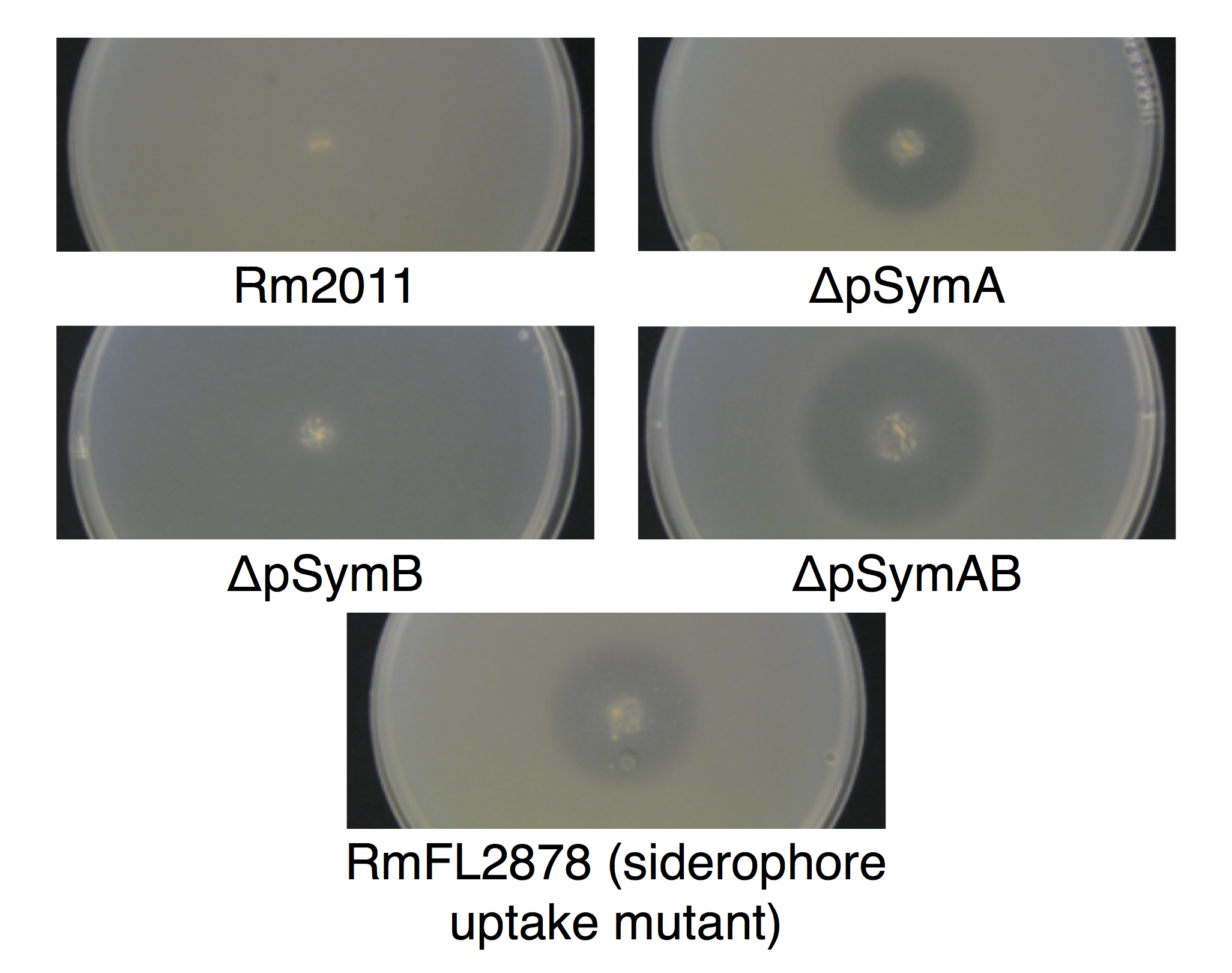

Supplement: Figure S2 — Images showing the bacteriocin-like effect of the pSymA-encoded siderophore. In all images, the stabbed strain is the wild-type S. meliloti Rm5000 (60), which is a rifampicin resistant and streptomycin sensitive derivative of the same nodule isolate of S. meliloti Rm2011 (streptomycin resistant). The wild type is able to inhibit the growth of strains lacking pSymA, but not a strain lacking just pSymB. The sensitivity of the pSymA cured strain is conferred by the inability to uptake the siderophore, as is seen by the sensitivity of S. meliloti RmFL2878 (rhtA::pTH1522), which is a siderophore uptake mutant (59). (TIFF) [file pgen.1004742.s006.tiff]

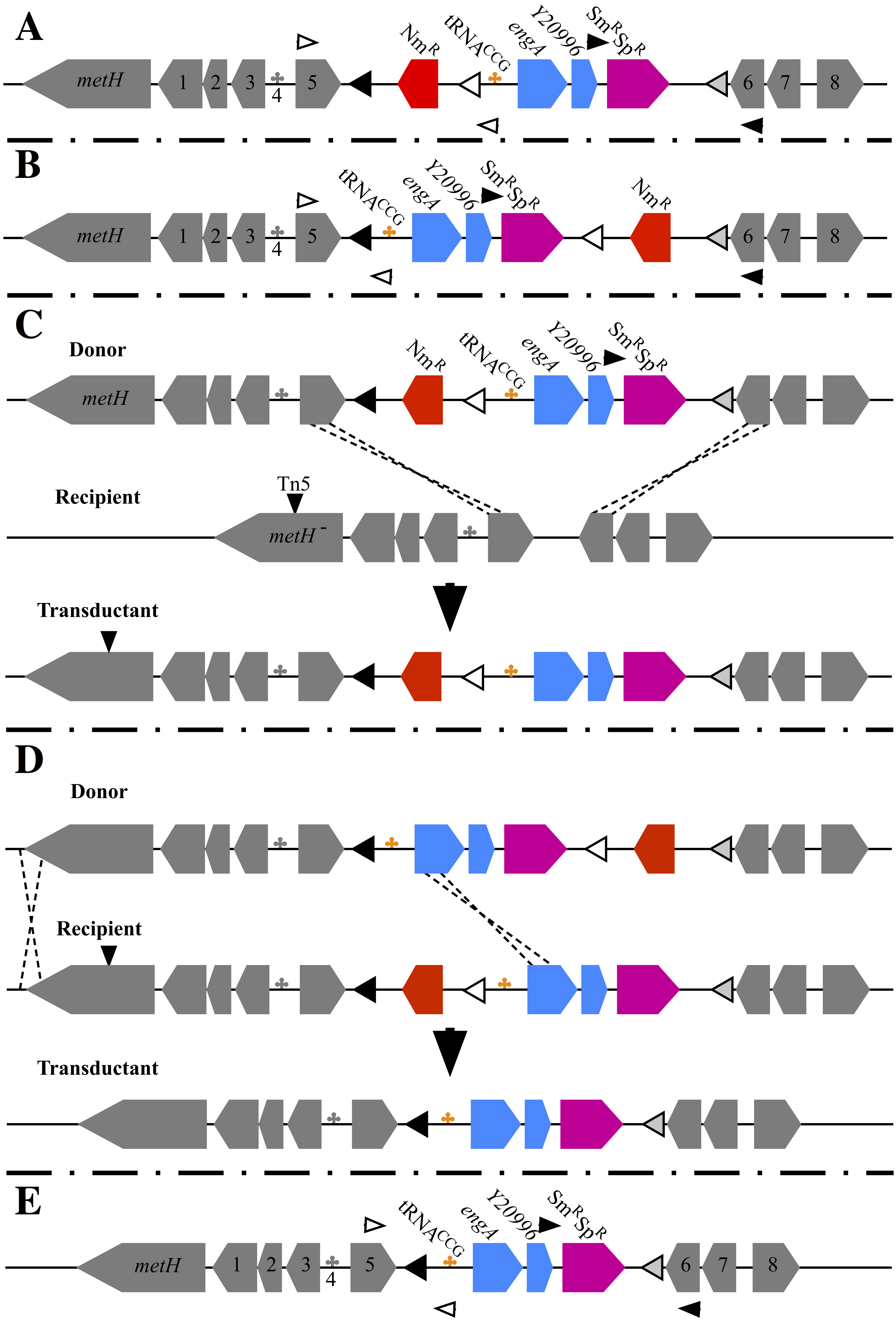

Supplement: Figure S3 — A diagrammatic representation of how the neomycin sensitive integration of the pSymB essential genes into the chromosome was constructed. (A) and (B) represent the two possible genetic organizations following integration into the chromosome, while (E) represents the final genetic organization. The arrows represent the approximate location of primers able to differentiate between each of the three organizations; the open-ended arrows will amplify a product in (B) and (E), while the close-ended arrows will amplify a product in (A) and (E). (C) and (D) illustrate the two transductions involved in the creation of a neomycin sensitive integration. The Tn5 insertion in metH is a loss-of-function mutation, rendering the strain unable to grow on minimal medium not supplemented with methionine. Diagrams are partially to scale. The attR sequence is indicated by the black arrowheads, the attL sequence by the light gray arrowheads, and the attP sequence by the white arrowheads. 1 – pmi; 2 – Y03111; 3 – mak; 4 – tRNACCT; 5 – Y03108; 6 – Y03107; 7 – Y03106; 8 – dxr. (TIFF) [file pgen.1004742.s007.tiff]
