# Supplementary material for: Examination of Prokaryotic Multipartite Genome Evolution through Experimental Genome Reduction
Source: PLoS Genet. 2014 Oct 23;10(10):e1004742. doi: 10.1371/journal.pgen.1004742 (PMC4207669; doi:10.1371/journal.pgen.1004742)
Supplement: Table S1 — Bacterial strains and plasmids. (DOCX) [file pgen.1004742.s008.docx]

| Strain or Plamid | | | Characteristics | | Resistance | Reference or Source |
| --- | --- | --- | --- | --- | --- | --- |
| *Sinorhizobium meliloti* | | | | |  |  |
|  | Rm1021 | Wild type SU47 *str-21* | | | Sm | [77] |
|  | Rm2011 | Wild type SU47 *str-3* | | | Sm | M. Hynes |
|  | Rm5000 | Wild type SU47 *rif-5* | | | Rif | [80] |
|  | SmA818 | Rm2011 cured of pSymA | | | Sm | [24] |
|  | RmFL2878 | RmP110, *rhtA*::pTH1522; siderophore uptake mutant | | | SmGm | [76] |
|  | RmFL2950 | RmP110, *rhbB*::pTH1522; siderophore biosynthesis mutant | | | SmGm | [76] |
|  | RmP110 | Rm1021 with wild type *pstC* | | | Sm | [78] |
|  | RmP798 | RmP110, ΔB122 (deletion of pSymBnt: 1,529,711 - 1,572,422), pTH1944 | | | Sm Nm GmTc | [28] |
|  | RmP801 | RmP110, ΔB116 (deletion of pSymBnt: 1,256,503 - 1,307,752), pTH1944 | | | SmTc | [28] |
|  | RmP806 | RmP110, ΔB123 (deletion of pSymBnt: 1,529,711 - 1,652,588), pTH1944 | | | Sm Nm GmTc | [28] |
|  | RmP963 | RmP110, ΔA133 (deletion of pSymAnt: 1,281,754 - 1,348,238), pTH1944 | | | Sm Nm GmTc | [28] |
|  | RmP1615 | *metH*::Tn5; methionine auxotroph | | | Sm Nm | Laboratory Collection |
|  | RmP1815 | RmP110, ΔB123 from RmP806 | | | Sm Nm Gm | This study |
|  | RmP2686 | RmP110, *attB*::(pTH2750) *smb20996-engAsmb21712* (tRNAarg gene) via *attP1* | | | SmSp Nm | [27] |
|  | RmP2711 | RmP110, *attB*::(pTH2750) *smb20996-engAsmb21712* (tRNAarg gene) via *attP2* | | | SmSp Nm | This study |
|  | RmP2719 | RmP110, *attR-smb21712-engA-smb20996-ΩSmSp-attL* via *attB* | | | SmSp | This study |
|  | RmP2745 | RmP110, ΔB180 (635,940 - 869,642), pTH1944 | | | Sm Nm Tc | [28] |
|  | RmP2778 | SmA818, *attR-smb21712-engA-smb20996-ΩSmSp-attL* from RmP2917 | | | SmSp | This study |
|  | RmP2805 | RmP2778, ΔB180 from RmP2745 | | | SmSp Nm | This study |
|  | RmP2917 | Rm2011 cured of pSymA and pSymB | | | SmSp | This study |
|  | RmP3004 | Rm2011, ΔB180 from RmP2745 | | | Sm Nm | This study |
|  | RmP3005 | RmP3004, *attR-smb21712-engA-smb20996-ΩSmSp-attL* from RmP2778 | | | SmSp Nm | This study |
|  | RmP3009 | Rm2011 cured of pSymB | | | SmSp | This study |
| Other | |  | |  |  |  |
|  | *Aspergillus species* | | | Isolated from alfalfa farm soil |  | This study |
|  | *Pseudomonas syringae*pv. tomato DC3000 | | | Wild type | Rif | R. Cameron |
|  | *Streptomyces coelicolor*M145 | | | Wild type |  | M. Elliot |
| Plamids | | |  |  |  |  |
|  | pRK600 | | pRK2013Nm^R^::Tn9, RK2 tra genes | | Cm | [37] |
|  | pTH1414 | | A pOT1 derivative expressing the pSymB*incα* incompatibility element | | Gm | [29] |
|  | pTH1522 | | A transcriptional reporter vector, pBR322 origin | | Gm | [59] |
|  | pTH1944 | | A pBBRmcs-3 derivative expressing *flp* | | Tc | [28] |
|  | pTH2750 | | A pUX19 derivative with *attP-ΩSmSp-smb20996-engA-smb21712-attP* | | SmSp Km | [27] |
| Sm – streptomycin; Sp – spectinomycin; Gm – gentamycin; Nm – neomycin; Km – kanamycin; Tc – tetracycline; Cm – chloramphenicol; Rif – rifampicin. | | | | | | |

**Table S1.Bacterial strains and plasmids.**
