# Supplementary material for: Examination of Prokaryotic Multipartite Genome Evolution through Experimental Genome Reduction
Source: PLoS Genet. 2014 Oct 23;10(10):e1004742. doi: 10.1371/journal.pgen.1004742 (PMC4207669; doi:10.1371/journal.pgen.1004742)
Supplement: Table S2 — Physiochemical properties of the soil used in this study. (DOCX) [file pgen.1004742.s009.docx]

**Table S2. Physiochemical properties of the soil used in this study.**

| Characteristics | Soil Sample | |
| --- | --- | --- |
|  | Not autoclaved^*^ | Autoclaved |
| Total C | 4.94 % dry | 4.20 % dry |
| Inorganic C | 1.53 % dry | 1.38 % dry |
| Organic C | 3.41 % dry | 2.82 % dry |
| pH | 7.6 | 7.5 |
| NH_4_-N | 46.2 mg/kg dry | 51.8 mg/kg dry |
| NO_3_-N | 2.16 mg/kg dry | 1.22 mg/kg dry |
| NO_2_-N | 0.042 mg/kg | 0.094 mg/kg |
| N | 0.35% | 0.30% |
| P | 146 mg/kg dry | 127 mg/kg dry |
| Mg | 494 mg/kg dry | NT^†^ |
| K | 383 mg/kg dry | NT |
| S | 0.05 % dry | 0.05 % dry |
| Ca | 2713 mg/kg dry | NT |
| Fe | 36.46 mg/kg dry | NT |
| Mn | 172.5 mg/kg dry | NT |
| Zn | 24.79 mg/kg dry | NT |
| % soil moisture | 20.64% | 20.65% |
| Organic matter | 5.9 % dry | 5.3 % dry |
| Very coarse sand | 0.8 % w/w | 0.5 % w/w |
| Coarse sand | 4.2 % w/w | 3.8 % w/w |
| Medium sand | 7.0 % w/w | 6.7 % w/w |
| Fine sand | 16.6 % w/w | 18.0 % w/w |
| Very fine sand | 21.2 % w/w | 22.1 % w/w |
| Sand | 49.8 % w/w | 51.1 % w/w |
| Silt | 35.4 % w/w | 34.3 % w/w |
| Clay | 14.8 % w/w | 14.6 % w/w |
| Texture | Loam | Loam |
| Organic matter | 5.9 % dry | 5.3 % dry |
| Gravel | 0.0 % w/w | 0.0 % w/w |

^*^Analysis performed following γ–irradiation of soil samples by the University of Guelph Laboratory Services Agricultural and Food Laboratory.

^†^NT, not tested.
